# MSC-derived exosomal miR-140-3p improves cognitive dysfunction in sepsis-associated encephalopathy by HMGB1 and S-lactoylglutathione metabolism

**DOI:** 10.1038/s42003-024-06236-z

**Published:** 2024-05-11

**Authors:** Ying Ma, Xingguo She, Yang Liu, Xian Qin

**Affiliations:** 1 Department of Transplant Surgery, The Third Xiangya Hospital, Central South University, 410013 Changsha, China; 2 Department of Pathology, The Third Xiangya Hospital, Central South University, 410013 Changsha, China; 3 Department of Gynaecology, The Third Xiangya Hospital, Central South University, 410013 Changsha, China

**Keywords:** Cell death in the nervous system, Infectious diseases, Cell signalling, Mesenchymal stem cells, RNA

## Abstract

MiRNAs in mesenchymal stem cells (MSCs)-derived exosome (MSCs-exo) play an important role in the treatment of sepsis. We explored the mechanism through which MSCs-exo influences cognitive impairment in sepsis-associated encephalopathy (SAE). Here, we show that miR-140-3p targeted *Hmgb1*. MSCs-exo plus miR-140-3p mimic (Exo) and antibiotic imipenem/cilastatin (ABX) improve survival, weight, and cognitive impairment in cecal ligation and puncture (CLP) mice. Exo and ABX inhibit high mobility group box 1 (HMGB1), IBA-1, interleukin (IL)-1β, IL-6, iNOS, TNF-α, p65/p-p65, NLRP3, Caspase 1, and GSDMD-N levels. In addition, Exo upregulates S-lactoylglutathione levels in the hippocampus of CLP mice. Our data further demonstrates that Exo and S-lactoylglutathione increase GSH levels in LPS-induced HMC3 cells and decrease LD and GLO2 levels, inhibiting inflammatory responses and pyroptosis. These findings suggest that MSCs-exo-mediated delivery of miR-140-3p ameliorates cognitive impairment in mice with SAE by HMGB1 and S-lactoylglutathione metabolism, providing potential therapeutic targets for the clinical treatment of SAE.

## Abstract (web summary)

MSCs-exo-mediated delivery of miR-1403p ameliorates cognitive impairment in mice with SAE by HMGB1-related microglial pyroptosis and S-lactoylglutathione metabolism.

## Introduction

Sepsis is a major clinical challenge that often leads to multiple organ dysfunction^1^. Sepsis-associated encephalopathy (SAE) is a serious complication of sepsis that may be present in up to 70% of patients with sepsis and increases mortality in patients with sepsis^2,3^. SAE is a multifactorial syndrome, characterized by diffuse cerebral dysfunction induced by the systemic response to infection without clinical or laboratory evidence of direct brain infection or other types of encephalopathy (e.g., hepatic or renal encephalopathy)^2,4^. However, effective clinical treatments for SAE are currently lacking. Determining the pathogenesis of SAE and identifying therapeutic targets are urgently needed.

High mobility group box 1 (HMGB1) is a major injury-associated molecular pattern that leads to fatal systemic inflammation and multiple organ dysfunction syndrome in critical diseases^5^. Studies have shown that HMGB1 mediates synaptic loss and cognitive impairment in animal models of SAE^6^. Berberine mitigates SAE by blocking the HMGB1/receptor for advanced glycation end products signaling pathway by inhibiting microglial stress on A1 astrocytes and neuron-induced decline^7^. In sepsis, the NOD-like receptor family pyrin domain containing 3 (NLRP3) inflammasome leads to the cleavage of Caspase 1 (the typical pathway) or Caspase 11 (the atypical pathway), thereby releasing interleukin (IL)-1β, IL-18, and HMGB1, and leading to pyroptosis^8^. Recombinant CC16 reduces NLRP3, Caspase 1, IL-6, and IL-1β levels, and the phosphorylation of p38 MAPK and extracellular-signal-regulated kinase (ERK), thereby inhibiting cortical pyroptosis and ameliorating SAE^9^. The role of HMGB1 in SAE is well understood, but the underlying molecular mechanism through which HMGB1 mediates microglial pyroptosis is poorly understood.

Mesenchymal stem cells (MSCs) are pluripotent stromal cells that can be isolated from various biological tissues, including adult bone marrow, adipose tissue, and neonatal tissue^10^. In mice with SAE, MSCs treatment can protect the integrity of the blood‒brain barrier, reduce astrogliosis and neuroinflammation, and improve cognition and behavior^11^. Our previous research confirmed that MSCs could improve the cognitive function of mice with SAE^12^, but the specific mechanism of action is still unknown. MSCs-derived exosomes (MSCs-exo) are new candidates for cell-free therapy for various diseases^13^. MSCs-exo carries long noncoding RNAs, microRNAs (miRNAs), messenger RNAs (mRNAs), and proteins, enabling them to have profound effects on recipient cells through epigenetic regulation^14^. MSCs and MSC-derived conditioned media alleviate SAE by reducing inflammation and inhibiting the ERK pathway^15^. The plasma exosome-derived long noncoding RNA NEAT1 promotes ferroptosis by regulating the miR-9-5p/transferrin receptor and the GOT1 axis, thereby exacerbating SAE^16^. MiR-146a-5p delivery by MSCs-exo protects the cardiomyocytes and myocardial tissues during sepsis by regulating MYBL1^17^. MSCs-exo alleviates M1 microglial activation in mice with brain injury caused by subarachnoid hemorrhage by delivering miR-140-5p^18^. MiR-140-3p inhibits the progression of sepsis by reducing the expression of genes associated with inflammation and apoptosis^19^. MiR-140-3p alleviates inflammation, oxidative stress, and apoptosis caused by oxygen–glucose deprivation and reperfusion, thereby exacerbating ischemia–reperfusion-induced brain injury^20^. MiR-140-3p protects hippocampal neuronal cells from pyroptosis and alleviates post-operative cognitive dysfunction induced by sevoflurane inhalation in rats^21^. In addition, miR-140-3p, which targets HMGB1, ameliorates the inflammatory response in airway smooth muscle cells by regulating the JAK2/STAT3 signaling pathway^22^. Bone marrow MSCs (BMSCs)-derived exosomes inhibit hypophosphorous-induced aortic calcification and improve renal function through the SIRT6-HMGB1 deacetylation pathway^23^. However, whether MSCs-exo affects cognitive impairment in SAE through HMGB1-associated microglial pyroptosis and the molecular mechanisms are unclear.

Human microglial clone 3 (HMC3) cells were established by simian virus (SV40)-dependent immortalization of human microglia in Professor Tardieu’s laboratory in 1995^24,25^ and have been widely used in studies related to brain diseases^26–28^. This study explored whether MSCs-exo regulated HMGB1-related pyroptosis and S-lactoylglutathione metabolism in HMC3 cells, thereby affecting cognitive dysfunction during SAE and the potential molecular mechanisms to provide insights for the use of anti-HMGB1 therapy to treat SAE.

## Methods

### Identification of differentially expressed microRNAs (DEmiRNAs)

The GSE101639 dataset included three normal controls, three patients with sepsis, and three patients with septic shock and was used to analyze DEmiRNAs associated with SAE. The expression matrix was normalized using the quantile method through the limma package. A |log_2_FoldChange| > 1 and adjusted *P* < 0.05 were used to identify DEmiRNAs. TargetScan (http://www.targetscan.org/mamm_31/) was used to predict the miRNAs targeting the combination of HMGB1. The intersection was used to obtain 15 prediction target miRNAs for subsequent research.

### Cell culture and intervention

The methods used to extract and identify MSCs and MSCs-exo, and other protocols are shown in Supplementary Methods. Human microglia HMC3 cells (AW-CNH003, Abiowell, Changsha, China) were cultured in minimum essential medium (MEM) supplemented with 10% fetal bovine serum, 100 IU/mL penicillin, and 10 μg/mL streptomycin at 37 °C, 5% CO_2_. The cells were randomly divided into the normal group, LPS group, MSCs-exo group, and MSCs-exo + miR-140-3p mimics group. Then, 100 ng/mL lipopolysaccharide (LPS) was added and incubated for 4 h under standard culture conditions^29^. MSCs-derived exosome (MSCs-exo) (1 μg/mL)^30^ or S-lactoylglutathione (10 mM)^31^ were with the LPS. The MSCs-exo + miR-140-3p mimics group consisted of MSCs-exo transfected with miR-140-3p mimics (1 μg/mL).

### Dual-luciferase reporter assay

TargetScan (http://www.targetscan.org/mamm_31/) was used to predict the binding sites of miR-140-3p and Hmgb1. Mutant (mut-Hmgb1) and wild-type (wt-Hmgb1) sequences were designed, synthesized according to the predicted results, cloned, and inserted into the pmirGLO reporter vector (Synthgene Biotech, Nanjing, China). The vector was transfected into HEK293T cells (AW-CNH086, Abiowell) with miR-140-3p mimics or mimics-NC (HonorGene, Changsha, China). Then, luciferase activity was determined by a dual-luciferase reporter gene assay kit (Promega, Shanghai, China). Renilla luciferase activity was used as an internal control.

### Animal experiment

C57BL/6J male mice, which were aged 6–8 weeks and weighed 25–30 g, were purchased from Hunan Slack Jingda Laboratory Animal Co., Ltd. The IRB of Third Xiangya Hospital, Central South University, approved all the experiments in this study (2023-S633). The experiments complied with all relevant ethical regulations for animal use. The mice were randomly divided into five groups (*n* = 10): the sham group, model group, MSCs-exo + miR-140-3p mimic (Exo) group, antibiotic (ABX) group, and ABX + Exo group. The cecal ligation puncture (CLP) model was constructed^12^. In brief, animals were anesthetized with 2% sevoflurane in a well-ventilated room. The distal cecum was ligated 40% from the base and pierced once with a 20-G needle. The sham group underwent the same laparotomy but not CLP. The remaining groups were used to construct CLP models. Bupivacaine (3 mg/kg) and buprenorphine (0.1 mg/kg) were injected subcutaneously to avoid postoperative pain. On the 8th day after surgery, 200 μg of exosomes were injected through the tail vein^32^. For five consecutive days after CLP, 25 mg/kg/day of imipenem/cilastatin and 0.9% NaCl were injected intraperitoneally once per day^33^. The mice were continuously observed for 25 days and killed on the 31st day. The whole brain was dissected, rinsed with precooled saline, and fixed in 4% paraformaldehyde for paraffin-embedded sections or further dissected for the hippocampus tissue. Then, the dissected hippocampus tissue was snap-frozen by liquid nitrogen and stored at −80 °C for subsequent molecular biological detection (*n* = 3) and metabolites analysis (*n* = 3).

### Morris water maze

As previously described^12^, the mice were trained four times daily for 5 days. After pretraining, the mice were tested at intervals of 5 s, 20 min, and 2 h (the interval between trials 1 and 2). Each test session lasted for 3 days. The time/path length was calculated by subtracting Trial 2 time/path length from that of Trial 1. A larger time/path length difference indicated better performance.

### Immunohistochemistry

Hippocampal tissue was fixed with 4% paraformaldehyde and cut into 2 μm slices. The slices were baked at 62 °C for 8 h, dewaxed, and rehydrated. The slices were soaked in 0.01 M citrate buffer at high temperature for 20 min and placed in a periodate solution to inactivate endogenous enzymes. The slices were incubated with primary antibodies against Caspase 1 (1:300, 22915-1-AP, Proteintech, USA) and NLRP3 (1:300, 19771-1-AP, Proteintech, USA) at 4 °C overnight. The slices were incubated with HRP-conjugated polyclonal goat anti-rabbit IgG (AWS0002, Abiowell, China). The slices were observed and analyzed under a microscope (BA210T, Motic, Singapore).

### Immunofluorescence analysis

The slices were subjected to immunohistochemical staining. After the cells were fixed with 4% paraformaldehyde for 30 min, they were permeabilized with 0.3% Triton X-100 for 30 min. Then, the slices were blocked with 5% bovine serum albumin (BSA) at 37 °C for 90 min. The slices were incubated with primary antibodies against Caspase 1 (1:300, 22915-1-AP, Proteintech, USA), NLRP3 (1:300, 19771-1-AP, Proteintech, USA), HMGB1 (1:200, ab190377, Abcam, UK) and ionized calcium-binding adaptor molecule-1 (IBA-1) (1:50, 10904-1-AP, Proteintech, USA) at 4 °C overnight. The slices were incubated with secondary antibodies at 37 °C for 60 min. The slices were then preserved in glycerin buffer and observed under fluorescence microscopy.

### Quantitative reverse transcription polymerase chain reaction (qRT-PCR)

Total RNA was extracted from cells and reverse-transcribed into complementary DNA (cDNA) using TRIzol reagent (15596026, Thermo Fisher Scientific, MA, USA) and a reverse transcription kit (CW2141, CWBIO, Beijing, China). PCR was performed using UltraSYBR mixture (CW2601, CWBIO). U6 was used as the internal control for miRNA expression. Relative miRNA expression was determined by the 2^−ΔΔCt^ method. The primer sequences are shown in Supplementary Table 1.

### Western blot analysis

Total proteins were extracted from brain tissue and neuronal cells with RIPA lysis buffer (AWB0136, Abiowell). The bicinchoninic acid (BCA) method was used to determine the protein concentration (AWB0104, Abiowell). The proteins were separated by 10% sodium dodecyl sulfate‒polyacrylamide gel electrophoresis and transferred to nitrocellulose membranes (AWB0231, Abiowell). The membranes were blocked in 5% skim milk at room temperature for 2 h and incubated with the primary antibodies shown in Supplementary Table 2. The membrane was incubated with secondary antibody HRP Goat anti-mouse IgG (SA00001-1, 1:5000, Proteintech, USA) or HRP Goat anti-rabbit IgG (SA00001-2, 1:6000, Proteintech, USA) at room temperature for 90 min. A ChemiScope6100 system (CLiNX) was used to capture the chemiluminescence signals from the membrane and produce a digital image of the protein bands. The band density in the image was then quantified using ImageJ software (ImageJ 1.5, USA). Uncropped blot images are shown in Supplementary Fig. 1.

### Statistics and reproducibility

The data were analyzed and plotted using GraphPad Prism 9 software. The data are expressed as the mean ± standard deviation (SD). Each test was repeated independently three times from distinct samples. Kolmogorov–Smirnov test and exploratory descriptive statistics test were used to analyze whether the data conformed to a normal distribution and homogeneity of variance. The measurement data obeyed the normal distribution and homogeneity of variance. The data were analyzed by parametric test. The unpaired Student’s *t*-test was used to compare the data of two groups that were not one-to-one correspondence using a two-tailed approach. One-way ANOVA and Tukey’s post-hoc test were used to compare data among three groups. Data comparisons between groups at different time points were analyzed by two-way ANOVA with Bonferroni as a post hoc test. The significance level was set at *P* < 0.05.

### Reporting summary

Further information on research design is available in the Nature Portfolio Reporting Summary linked to this article.

## Results

### Bioinformatics analysis of HMGB1-targeting miRNAs in clinical SAE

**Fig. 1:**
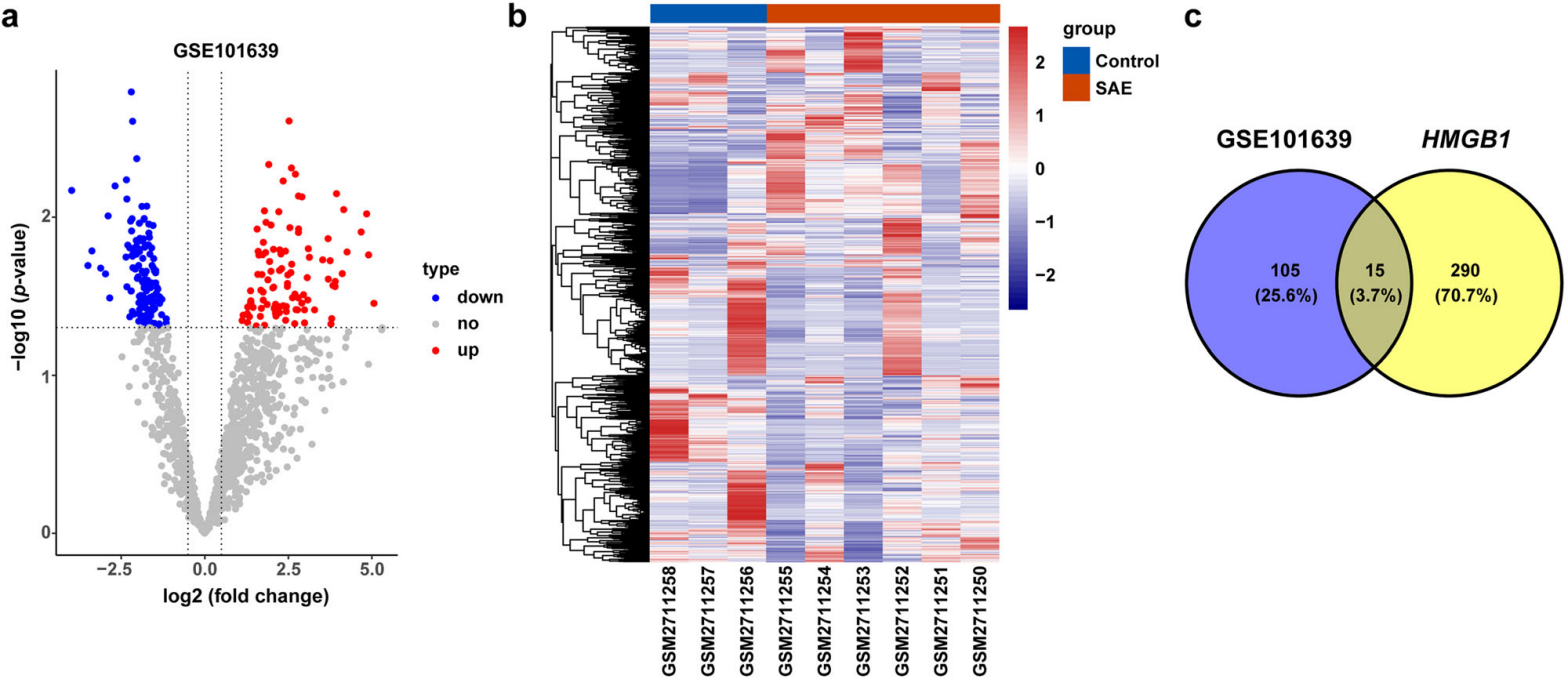
MiRNAs targeting HMGB1 in SAE. **a** Volcano map showing DEmiRNAs in the control group and SAE group. **b** Heatmaps showing the variations in miRNA abundance. **c** Venn diagram showing the intersection of DEmiRNAs and HMGB1-targeting miRNAs.

We first identified DEmiRNAs in SAE patients in the GSE101639 dataset. Volcano maps were constructed for the DEmiRNAs in the control and SAE groups (Fig. 1a). The heatmap shows variations in miRNA abundance (Fig. 1b). The Venn diagram shows that the DEmiRNAs and *HMGB1* targeting miRNAs shared 15 common miRNAs: hsa-miR-46445-5p, hsa-miR-378b, hsa-miR-642a-3p, hsa-miR-4275, hsa-miR-548as-3p, hsa-miR-611, hsa-miR-142-3p, hsa-miR-361-3p, hsa-miR-450a-5p, hsa-miR-589-5p, hsa-miR-140-3p, hsa-miR-147a, hsa-miR-4733-3p, hsa-miR-205-5p, and hsa-miR-340-5p (Fig. 1c).

### miR-140-3p was highly expressed in MSCs-exo

**Fig. 2:**
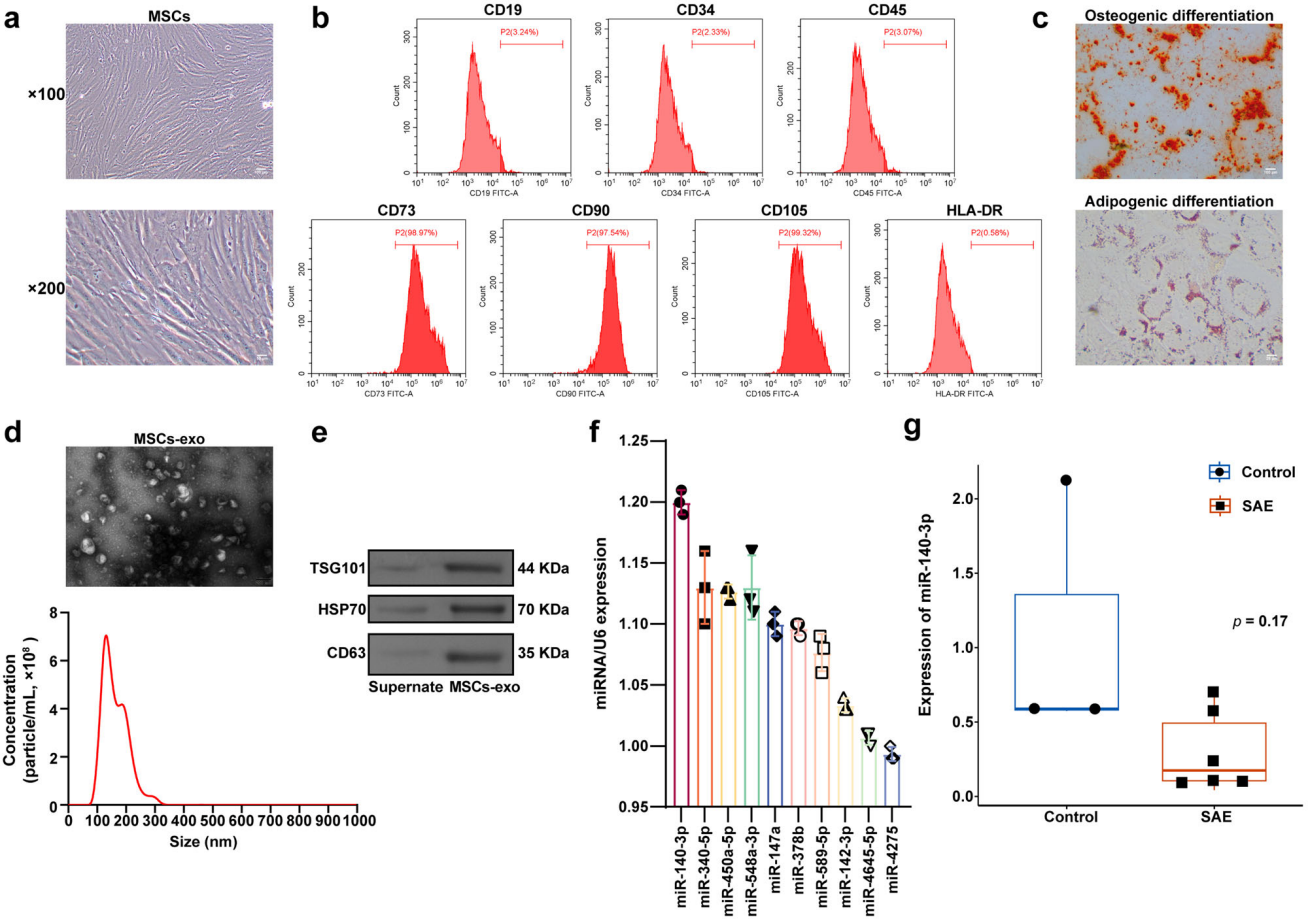
Verification of the expression of HMGB1-targeting miRNAs in MSCs-exo. **a** Morphological observation of MSCs. Scale bars: ×100 images, 100 µm; ×200 images, 50 µm. **b** Flow cytometric analysis of MSC surface markers. **c** Bone and lipid-induced differentiation of MSCs was observed by alizarin red staining and oil red O staining, respectively. Scale bars: ×100 images, 100 µm; ×400 images, 25 µm. **d** TEM and NTA were used to observe the morphology of exosomes derived from MSCs. Scale bars: 200 nm. **e** Western blot analysis of exosome surface markers (TSG101, HSP70, and CD63). **f** The expression of the top 10 miRNAs in MSCs-exo was measured by qRT-PCR. Each experiment was independently repeated three times. **g** The box diagram showing the miR-140-3p level in the control (*n* = 3) and SAE (*n* = 6) groups.

First, the morphology of the MSCs was observed by optical microscopy. The results showed that MSCs grew like fibrocytes, with spindle-shaped or irregular triangles on the surface of the support, oval-shaped nuclei in the center of the cells, and protrusions of different lengths in the cytoplasm. Almost all the MSCs were adherent and had strong adherent abilities (Fig. 2a). The flow cytometry results showed that cluster designation (CD)73 (98.97%), CD90 (97.54%), and CD105 (99.32%) were highly expressed. The expression levels of CD19 (3.24%), CD34 (2.33%), CD45 (3.07%), and human leukocyte antigen-DR isotype (HLA-DR) (0.58%) were low (Fig. 2b and Supplementary Fig. 2). The MSCs exhibited osteogenic differentiation and lipogenic differentiation ability (Fig. 2c). Our results indicated that the isolation of MSCs was successful. Transmission electron microscopy (TEM) revealed that the isolated exosomes were cup- or spherical-shaped, and the nanoparticle tracking analysis (NTA) results revealed that the diameters of the exosomes were ~30–150 nm (Fig. 2d), indicating the successful extraction of the exosomes. Exosome surface markers (TSG101, HSP70, and CD63) were examined by western blot. The results showed that TSG101, HSP70, and CD63 were highly expressed in MSCs-exo (Fig. 2e). The expression of the top 10 DEmiRNAs in MSCs-exo was measured by qRT-PCR. The results showed that the expression level of miR-140-3p was the highest (Fig. 2f). The GSE101639 dataset showed decreased miR-140-3p expression in SAE samples (Fig. 2g). Therefore, we further explored the role of MSCs-exo-mediated delivery miR-140-3p in SAE.

### MSCs-exo-mediated delivery of miR-140-3p inhibited LPS-induced HMC3 cell inflammation and pyroptosis

**Fig. 3:**
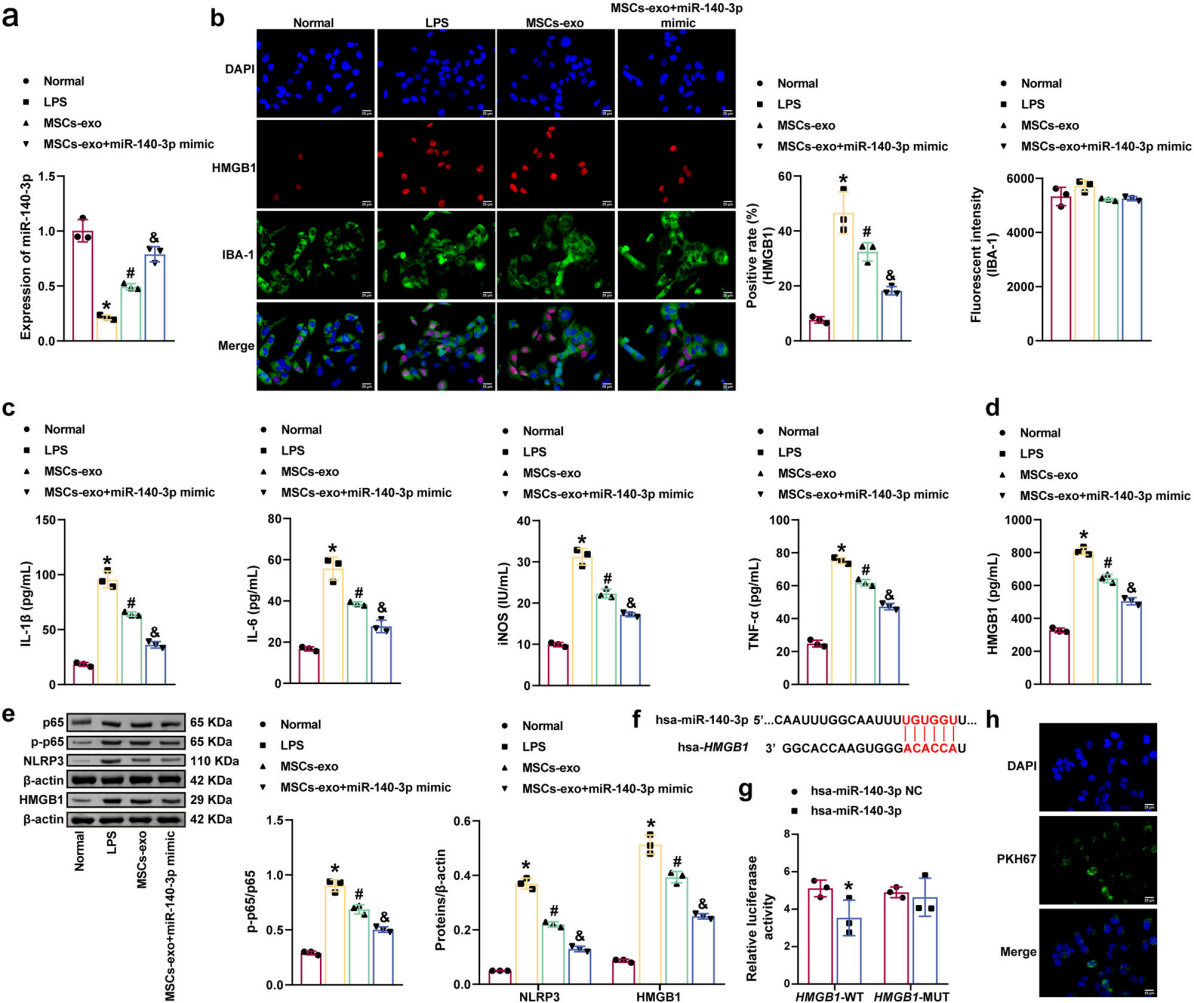
MSCs-exo-mediated delivery of miR-140-3p inhibited LPS-induced HMC3 cell inflammation and pyroptosis. **a** MiR-140-3p levels were measured by qRT-PCR. **b** IBA-1 and HMGB1 levels in HMC3 cells were examined by immunofluorescence analysis. Scale bars: 25 µm. **c**, **d** ELISA was used to examine iNOS, IL-1β, IL-6, TNF-α, and HMGB1 levels in the supernatant of HMC3 cells. **e** Western blot analysis of p65, p-p65, NLRP3, and HMGB1 expression. **f** Bioinformatics analysis was used to predict the binding sites of miR-140-3p to HMGB1 (human). **g** The dual-luciferase reporter assay confirmed the binding of miR-140-3p to HMGB1. **h** The uptake of MSCs-exo was observed by immunofluorescence analysis. Scale bars: 25 µm. Each experiment was independently repeated three times. Error bars show SD. *P < 0.05 vs. the normal group; #*P* < 0.05 vs. the LPS group; &*P* < 0.05 vs. the MSCs-exo group.

The effects of miR-140-3p delivery by MSCs-exo effects on LPS-induced HMC3 cell inflammation and pyroptosis were further investigated. MSCs-exo increased miR-140-3p levels in LPS-induced HMC3 cells. MiR-140-3p expression was increased in the MSCs-exo + miR-140-3p mimic group compared with the MSCs-exo group (Fig. 3a). LPS increased HMGB1 levels in HMC3 cells. MSCs-exo decreased HMGB1 levels in LPS-induced HMC3 cells. MSCs-exo + miR-140-3p mimic further inhibited HMGB1 levels in LPS-induced HMC3 cells (Fig. 3b). MSCs-exo decreased inducible nitric oxide synthase (iNOS), IL-1β, IL-6, tumor necrosis factor-α (TNF-α), and HMGB1 levels in LPS-induced HMC3 cells. Treatment with MSCs-exo and the miR-140-3p mimic further reduced iNOS, IL-1β, IL-6, TNF-α, and HMGB1 levels in LPS-induced HMC3 cells (Fig. 3c, d). In addition, MSCs-exo reduced the p-p65/p65 ratio and NLRP3 levels in LPS-induced HMC3 cells. Furthermore, the p-p65/p65 ratio and NLRP3 and HMGB1 levels were further reduced in the MSCs-exo + miR-140-3p mimic group (Fig. 3e). Bioinformatics analysis revealed the binding sites of miR-140-3p and HMGB1 (human). A dual-luciferase reporter assay confirmed the binding of miR-140-3p to HMGB1 (Fig. 3f, g). Moreover, fluorescence microscopy revealed that HMC3 cells internalized the MSCs-exo (Fig. 3h). Our findings suggested that the delivery of miR-140-3p by MSCs-exo reduced LPS-induced HMC3 cell inflammation and pyroptosis.

### MSCs-exo-mediated delivery of miR-140-3p improved cognitive impairment in mice with SAE

**Fig. 4:**
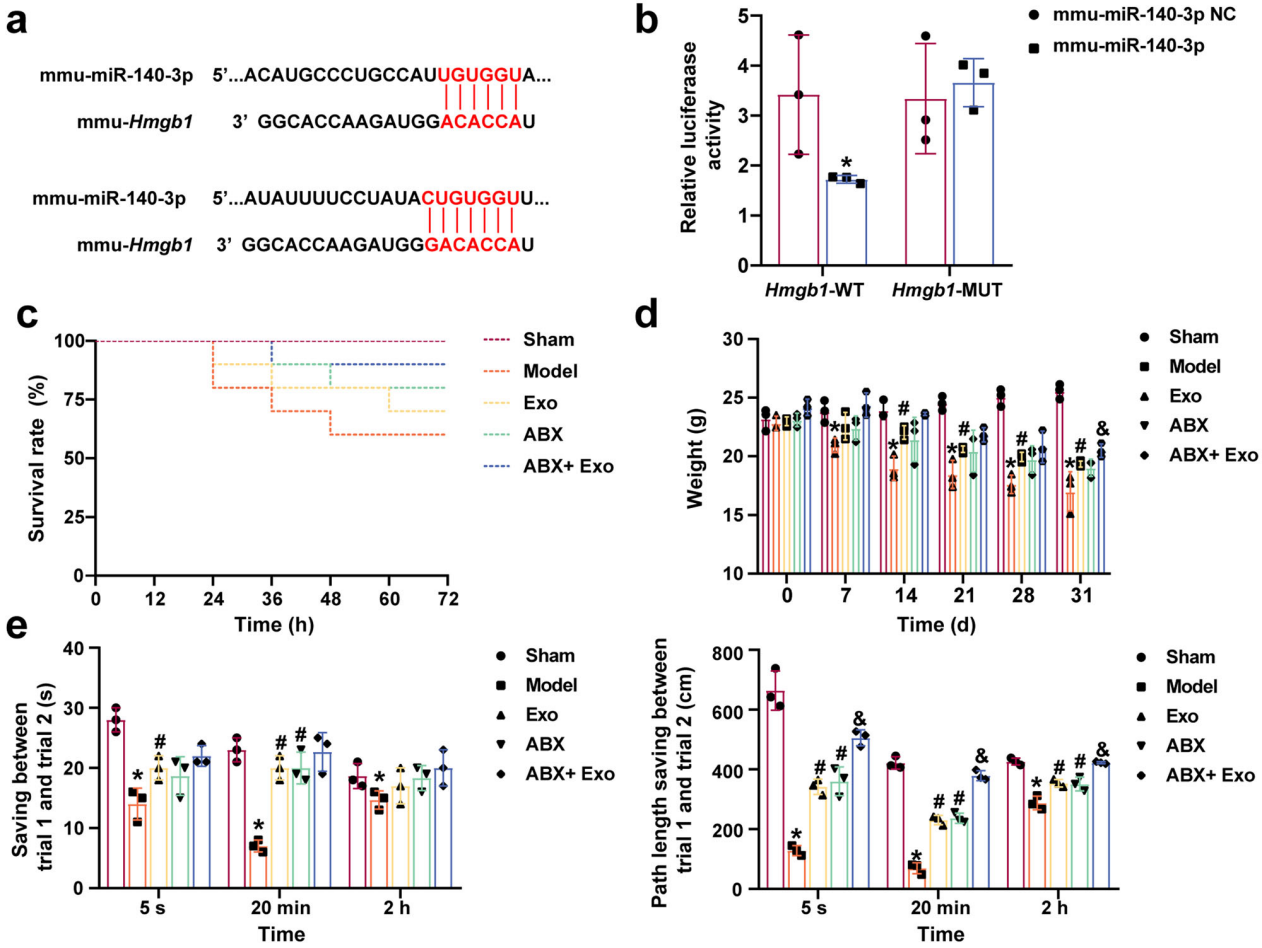
MSCs-exo-mediated delivery of miR-140-3p improved cognitive impairment in mice with SAE. **a** Bioinformatics analysis was used to predict the binding sites of miR-140-3p to *Hmgb1* (mouse). **b** The dual-luciferase reporter assay confirmed the binding of miR-140-3p to *Hmgb1*. **c** Survival rate analysis of CLP mice. **d** Changes in the weight of CLP mice. **e** Morris water maze test. *n* = 10. Error bars show SD. *P < 0.05 vs. the sham group; #*P* < 0.05 vs. the model group; & *P* < 0.05 vs. the ABX group.

Bioinformatics analysis revealed the binding site of miR-140-3p to Hmgb1 (mouse) (Fig. 4a). A dual-luciferase reporter assay confirmed the binding of miR-140-3p to Hmgb1 (Fig. 4b). Exo and ABX improved the survival rate of CLP model mice. The survival rate of mice in the ABX + Exo group further increased (Fig. 4c). Exo and ABX increased the body weight of model mice. The body weights of the mice in the ABX + Exo group were further increased (Fig. 4d). The Morris water maze test results showed that Exo and ABX improved the path length savings between trial 1 and trial 2 in CLP model mice. Path length savings between trial 1 and trial 2 for CLP model mice in the ABX + Exo group were further increased (Fig. 4e). Our results suggested that delivery of miR-140-3p by MSCs-exo and ABX improved cognitive impairment in mice with SAE, and ABX and MSCs-exo had synergistic effects.

### MSCs-exo-mediated delivery of miR-140-3p inhibited neuroinflammation and pyroptosis in mice with SAE

**Fig. 5:**
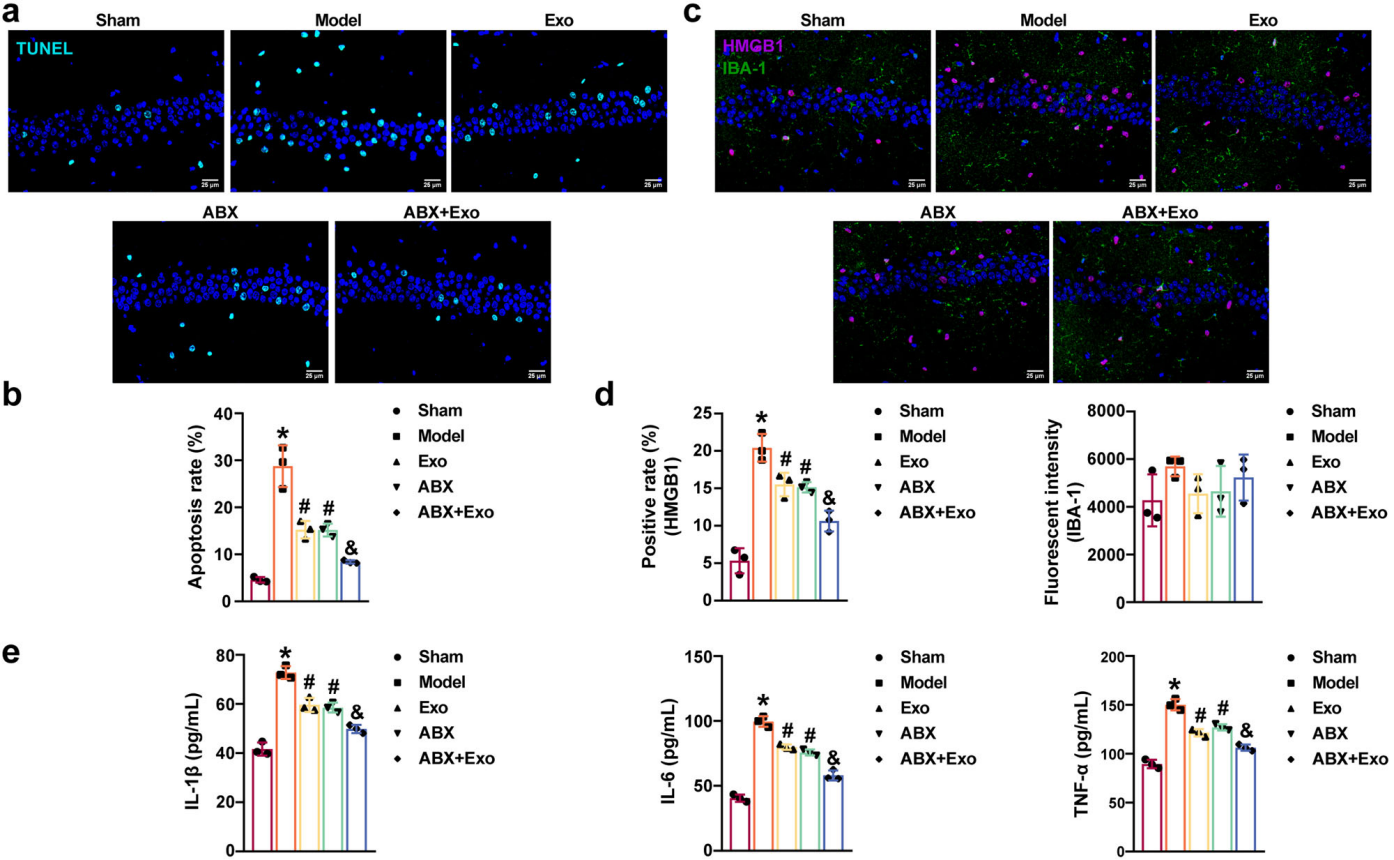
MSCs-exo-mediated delivery of miR-140-3p reduced neuroinflammation in mice with SAE. **a**, **b** Hippocampal apoptosis was observed by TUNEL staining. Scale bars: 25 µm. **c**, **d** IBA-1 and HMGB1 levels in hippocampal tissues were examined by immunohistochemistry. Scale bars: 25 µm. **e** ELISA was used to measure IL-1β, IL-6, and TNF-α levels in the hippocampus. *n* = 3. Error bars show SD. *P < 0.05 vs. the sham group; #*P* < 0.05 vs. the model group; & *P* < 0.05 vs. the Exo group.

**Fig. 6:**
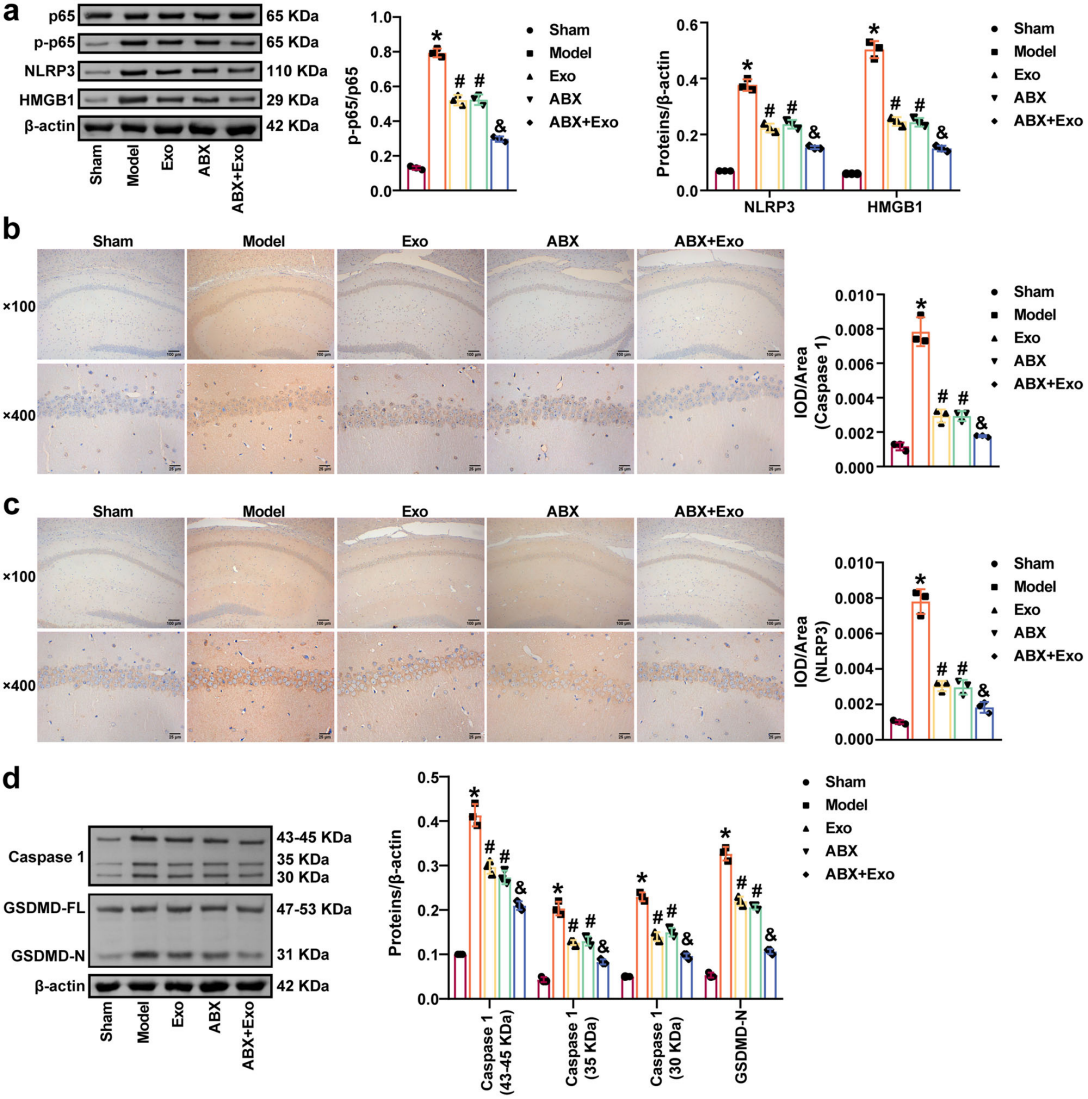
MSCs-exo-mediated delivery of miR-140-3p reduced pyroptosis in mice with SAE. **a** Western blot analysis of HMGB1, p65, p-p65, and NLRP3 expression in the hippocampus. **b**, **c** NLRP3 and Caspase 1 levels in the hippocampus were examined by immunohistochemistry. Scale bars: ×100 images, 100 µm; ×400 images, 25 µm. **d** Western blot analysis of Caspase 1 and GSDMD levels in the hippocampus. *n* = 3. Error bars show SD. *P < 0.05 vs. the sham group; #*P* < 0.05 vs. the model group; & *P* < 0.05 vs. the Exo group.

TUNEL staining showed CLP-induced apoptosis in the hippocampus in mice with SAE. Exo and ABX inhibited apoptosis in the hippocampus of CLP model mice, and ABX plus Exo further reduced apoptosis in the hippocampus of CLP model mice (Fig. 5a, b). The colocalization of IBA-1 and HMGB1 in the hippocampus was observed by immunofluorescence analysis. The CLP promoted the expression of IBA-1 and HMGB1 in the hippocampus. Exo and ABX decreased IBA-1 and HMGB1 levels in the hippocampus of CLP model mice, and ABX plus Exo further decreased IBA-1 and HMGB1 levels in the hippocampus of CLP model mice (Fig. 5c, d). Exo and ABX inhibited IL-1β, IL-6, and TNF-α levels in the hippocampus of CLP model mice, and ABX and Exo had a synergistic effect (Fig. 5e). Moreover, Exo and ABX decreased HMGB1, p-p65/p65, and NLRP3 levels in the hippocampus of CLP model mice. ABX plus Exo further inhibited HMGB1, p-p65/p65, and NLRP3 levels in the hippocampus of CLP model mice (Fig. 6a). In addition, Exo and ABX reduced NLRP3, Caspase 1, and GSDMD-N levels in the hippocampus of CLP model mice. Treatment with ABX plus Exo further inhibited NLRP3, Caspase 1, and GSDMD-N levels in the hippocampus of CLP model mice (Fig. 6b–d). Our findings revealed that delivery of miR-140-3p by MSCs-exo inhibited neuroinflammation and pyroptosis in mice with SAE.

### MSCs-exo-mediated delivery of miR-140-3p regulated NLRP3/Caspase 1-induced hippocampal tissue metabolism via HMGB1

**Fig. 7:**
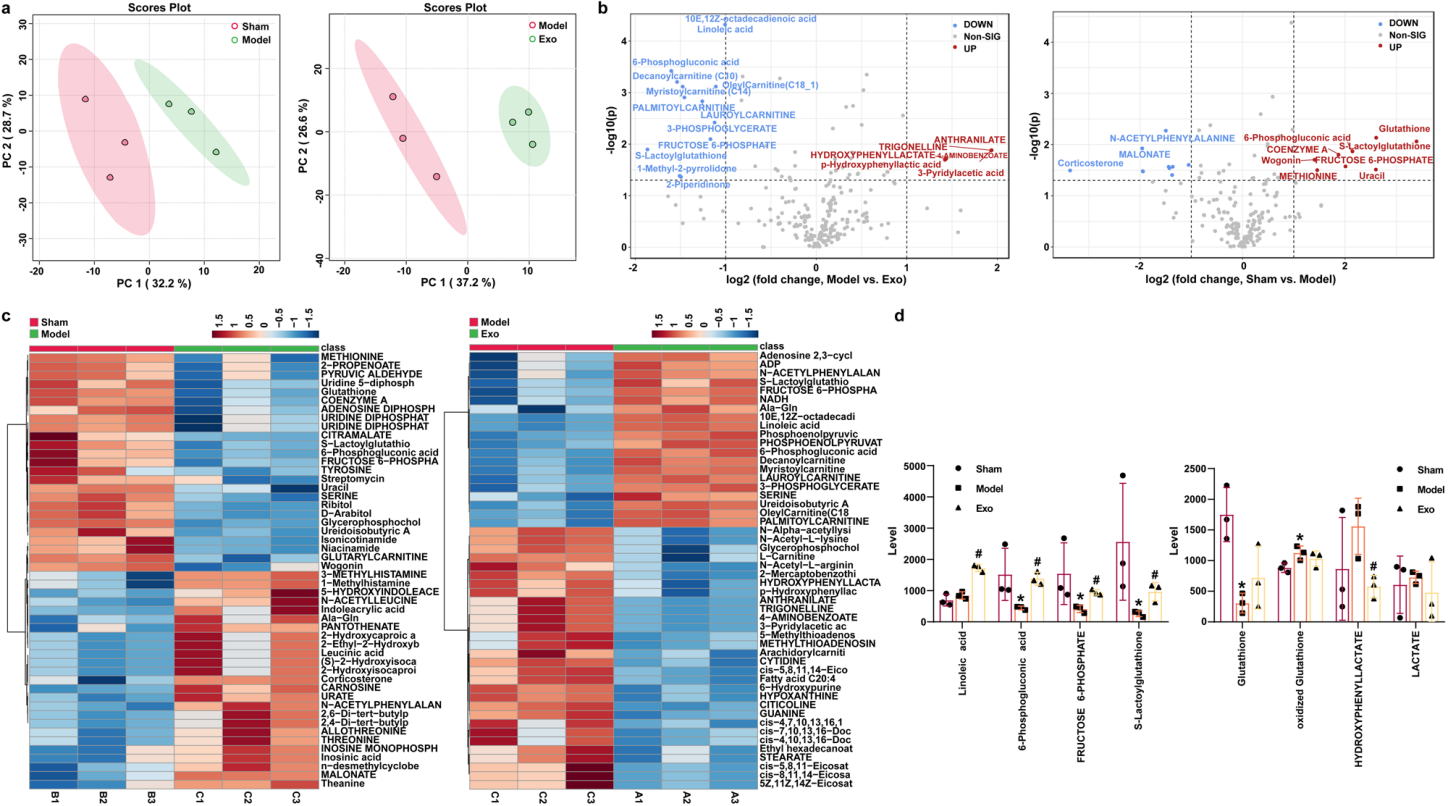
MiR-140-3p delivered by MSCs-exo changed the metabolic profile of the hippocampus of mice with SAE. **a** Principal component analysis. **b** Volcano plot showing differentially expressed metabolites in the groups. **c** Heatmap showing differences in metabolites between the groups. **d** Box plots showing key differentially expressed metabolites. Error bars show SD. *P < 0.05 vs. the sham group; #*P* < 0.05 vs. the model group.

**Fig. 8:**
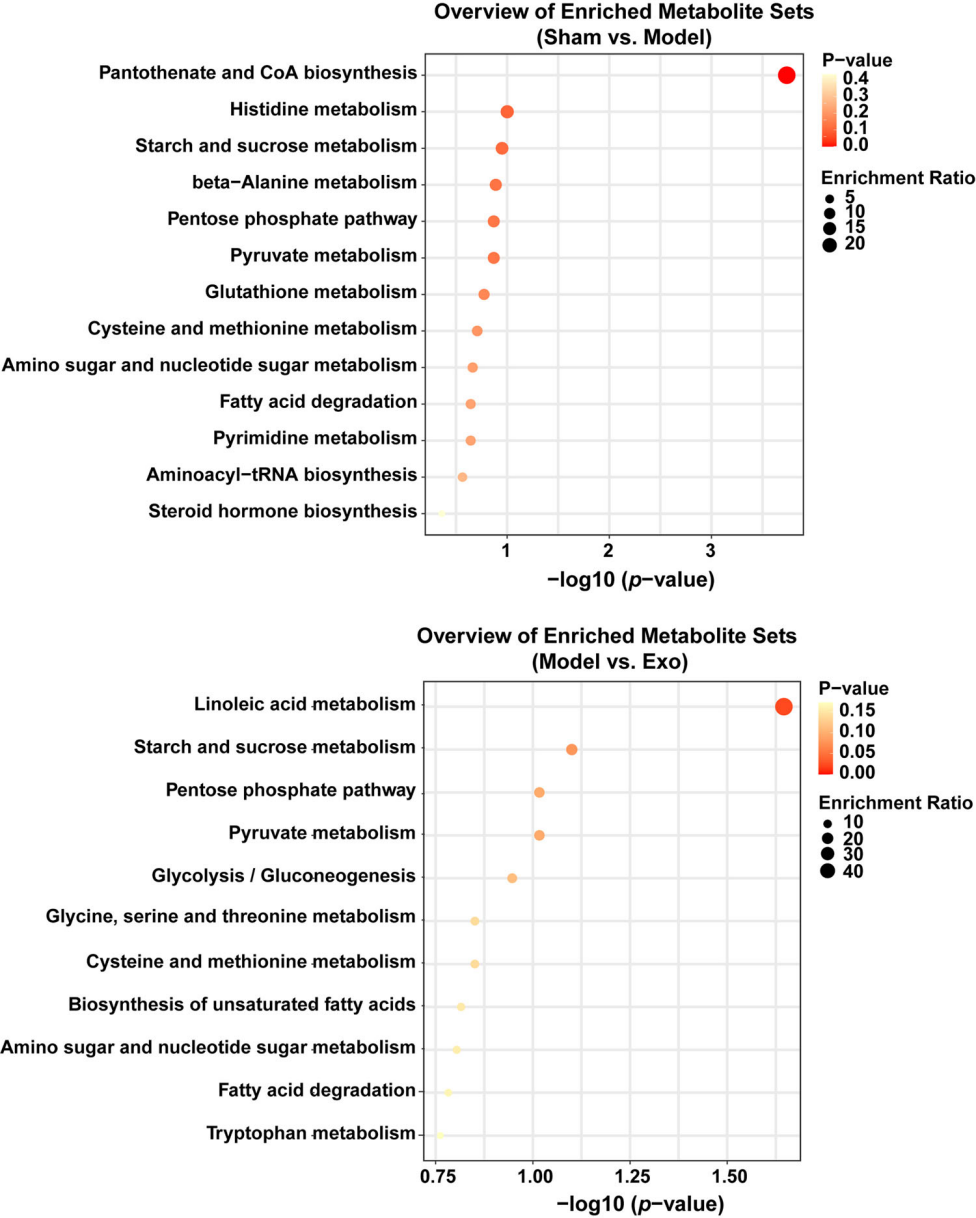
Overview of enriched metabolite sets between different groups. Effects of miR-140-3p delivered by MSCs-exo on metabolic function in the hippocampus of mice with SAE.

Next, LC–MS nontargeted metabolomics was performed to examine how the delivery of miR-140-3p by MSCs-exo affected metabolic reprogramming in the hippocampus of mice with SAE. Principal component analysis revealed differences in metabolic profiles between the sham, model, and Exo groups (Fig. 7a). Volcano plots and Heatmaps show differences in metabolites in hippocampal tissue between the different groups. The boxplot shows changes in key differentially expressed metabolites. Compared with those in the sham group, 6-phosphogluconic acid, fructose 6-phosphate, S-lactoylglutathione, and glutathione levels were lower in the model group, while the amount of oxidized glutathione was increased. Exo upregulated 6-phosphogluconic acid, fructose 6-phosphate, and S-lactoylglutathione levels in the model group (Fig. 7b–d). Enrichment analysis revealed that the differentially expressed metabolites regulated starch and sucrose metabolism, the pentose phosphate pathway, pyruvate metabolism, cysteine and methionine metabolism, amino sugar, and nucleotide sugar metabolism, and fatty acid degradation (Fig. 8). Our results suggested that Exo altered the metabolic characteristics of hippocampal tissue in mice with SAE.

### S-lactoylglutathione and miR-140-3p delivered by MSCs-exo could inhibit LPS-induced HMC3 cell inflammation and pyroptosis

**Fig. 9:**
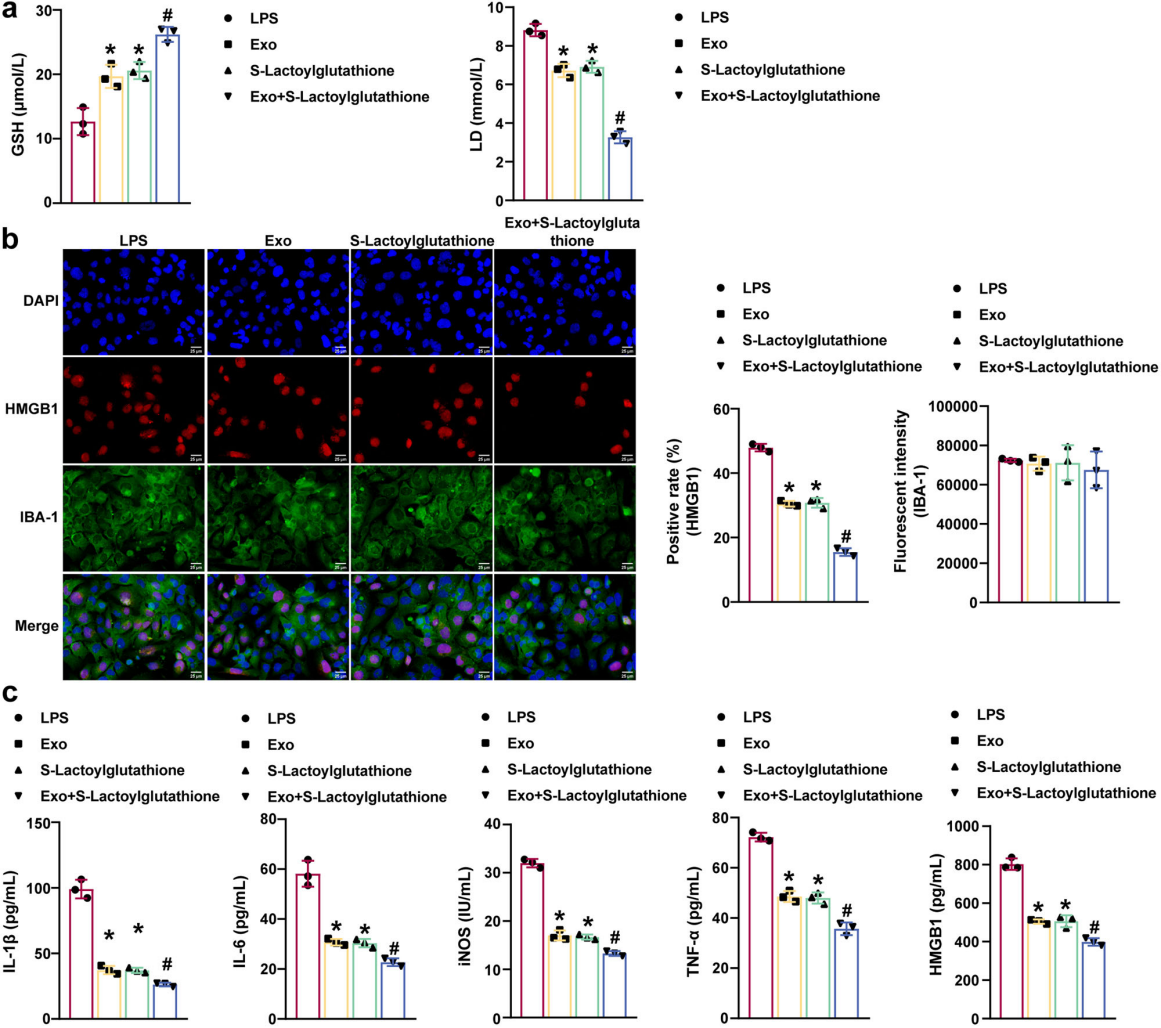
S-lactoylglutathione supplementation in combination with MSCs-exo delivery of miR-140-3p inhibited LPS-induced HMC3 cell inflammation. **a** GSH and LD levels were measured by ELISA. **b** IBA-1 and HMGB1 levels in HMC3 cells were examined by immunofluorescence analysis. Scale bars: 25 µm. **c** ELISA was used to measure iNOS, IL-1β, IL-6, TNF-α, and HMGB1 levels in the supernatant of HMC3 cells supernatant. Each experiment was independently repeated three times. Error bars show SD. *P < 0.05 vs. the LPS group; #*P* < 0.05 vs. the Exo group.

**Fig. 10:**
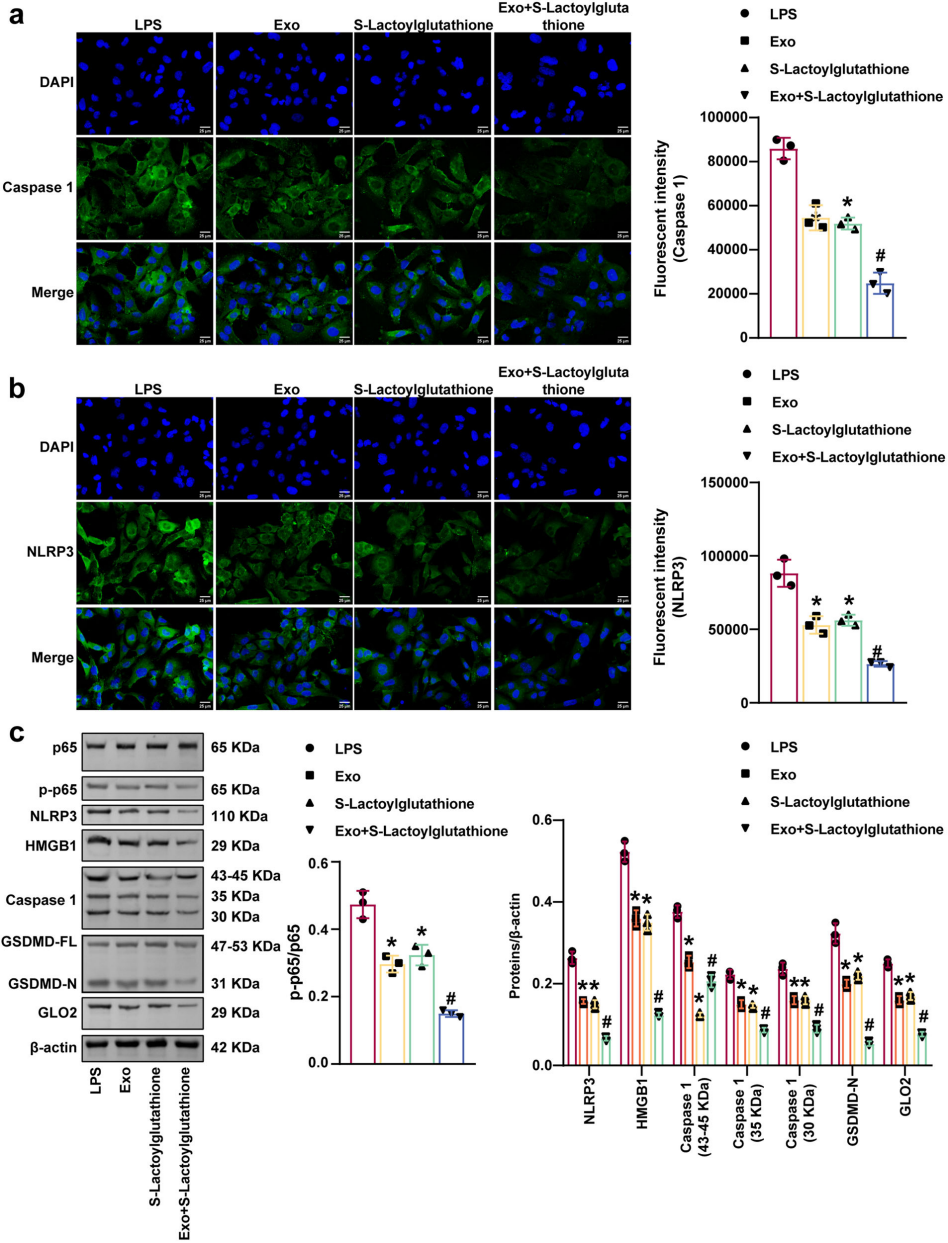
S-lactoylglutathione supplementation in combination with MSCs-exo delivery of miR-140-3p inhibited LPS-induced HMC3 cell pyroptosis. **a** and **b** NLRP3 and Caspase 1 expression were examined by immunofluorescence analysis. Scale bars: 25 µm. **c** Western blot analysis of HMGB1, GLO2, p65, p-p65, NLRP3, Caspase 1, and GSDMD levels. Each experiment was independently repeated three times. Error bars show SD. *P < 0.05 vs. the LPS group; #*P* < 0.05 vs. the Exo group.

The effects of S-lactoylglutathione on LPS-induced HMC3 cell activation and inflammasome expression were further explored and examined. ELISA results showed that Exo and S-lactoylglutathione promoted the production of glutathione (GSH) levels in LPS-induced HMC3 cells while inhibiting LD levels. Treatment with Exo plus S-lactoylglutathione further increased GSH levels in LPS-induced HMC3 cells and suppressed LD levels (Fig. 9a). Immunofluorescence analysis revealed that Exo and S-lactoylglutathione decreased IBA-1 and HMGB1 levels in HMC3 cells induced by LPS. Exo and S-lactoylglutathione further inhibited the LPS-induced changes in IBA-1 and HMGB1 levels in LPS-induced HMC3 cells (Fig. 9b). Exo and S-lactoylglutathione decreased iNOS, IL-1β, IL-6, TNF-α, and HMGB1 levels in LPS-induced HMC3 cells. Treatment with Exo plus S-lactoylglutathione further reduced iNOS, IL-1β, IL-6, TNF-α, and HMGB1 levels in LPS-induced HMC3 cells (Fig. 9c). In addition, the immunofluorescence results showed that Exo and S-lactoylglutathione reduced NLRP3 and Caspase 1 expression in LPS-induced HMC3 cells. NLRP3 and Caspase 1 were further decreased in the Exo + S-Lactoylglutathione group (Fig. 10a, b). The western blot results further demonstrated that Exo and S-lactoylglutathione downregulated HMGB1, GLO2, p-p65/p65, NLRP3, Caspase 1, and GSDMD-N levels in LPS-induced HMC3 cells. Treatment with Exo plus S-lactoylglutathione further inhibited HMGB1, GLO2, p-p65/p65, NLRP3, Caspase 1, and GSDMD-N expression (Fig. 10c). Our findings suggested that S-lactoylglutathione supplementation combined with the delivery of miR-140-3p by MSCs-exo to reduce LPS-induced HMC3 cell inflammation and pyroptosis.

## Discussion

In the present study, our results showed that MSCs-exo increased miR-140-3p expression in LPS-induced HMC3 cells. The expression of miR-140-3p was increased in the MSCs-exo + miR-140-3p mimic group compared with the MSCs-exo group. MSCs-exo-mediated delivery of miR-140-3p ameliorated cognitive impairment in CLP mice and inhibited neuroinflammation and pyroptosis. In addition, Exo increased S-lactoylglutathione levels in the hippocampal tissue of CLP mice. Our data further demonstrated that Exo and S-lactoylglutathione increased GSH content in LPS-induced HMC3 cells and inhibited LD and GLO2 levels, the inflammatory response, and pyroptosis.

MSCs are an attractive cell type for therapeutic exosome production^34^. Studies have shown that MSCs-exo alleviates sepsis-related acute liver injury by inhibiting MALAT1 through miR-26a-5p^35^. However, the role of MSCs-exo in SAE and the underlying mechanisms remain unclear. This study revealed that miR-140-3p expression was upregulated in MSCs-exo and downregulated in SAE. MiR-140-3p activates the 5-hydroxytryptamine receptor 2A/ERK/nuclear factor-E2-related factor 2 axis by targeting DNA methyltransferase 1, thereby protecting hippocampal neurons from pyroptosis and alleviating postoperative cognitive dysfunction caused by sevoflurane inhalation^21^. MiR-140-5p delivered by MSCs-exo reduces neuronal apoptosis and neuroinflammation by inhibiting the activation of M1 microglia, thus alleviating brain injury in rats with subarachnoid hemorrhage^18^. We hypothesized that MSCs-exo could influence SAE disease progression through miR-140-3p. To verify this hypothesis, we further explored the effect of MSCs-exo-mediated delivery of miR-140-3p on LPS-induced HMC3 cells and mice with SAE. Our results showed that MSCs-exo reduced IL-1β, IL-6, iNOS, TNF-α, and p65/p-p65 levels in LPS-induced HMC3 cells and in the hippocampus of CLP model mice. MSCs-exo improved survival, weight, and cognitive impairment in CLP model mice. Compared with MSCs-exo, MSCs-exo plus the miR-140-3p mimic further enhanced these effects. Studies have shown that intestinal epithelial exosomes inhibit IL-1β, IL-18, iNOS, and TNF-α expression in neuronal cells and ameliorate cognitive impairment in CLP model rats^36^. Our results are consistent with these studies. Our findings suggested that the delivery of miR-140-3p by MSCs-exo inhibited LPS-induced inflammatory responses in HMC3 cells and improved cognitive impairment in CLP model mice.

Eliminating the inflammatory cascade by HMGB1 may be a strategy to complement nonpharmacological interventions targeting SAE^37^. Inflachromene inhibits the expression of HMGB1 and IBA-1 in the hippocampus of CLP mice and restores cognitive function^6^. Berberine-mediated blockade of HMGB1/receptor for advanced glycation end products signaling reduces the expression of TNF-α, IL-1α, and complement C1q A chain in the hippocampus of CLP mice, inhibits the activation of microglia, and thus alleviates sepsis-induced cognitive impairment^7^. This work showed that MSCs-exo inhibited HMGB1 and IBA-1 expression in LPS-induced HMC3 cells and the hippocampal tissue of CLP mice. Overexpression of miR-140-3p enhanced this effect. A dual-luciferase reporter assay confirmed that miR-140-3p targeted HMGB1. Microglial pyroptosis-mediated neuroinflammation is critical for the pathogenesis of SAE. Studies have shown that Erbin downregulates NLRP3, Caspase 1, GSDMD-N, IL-1β, IL-18, and TNF-α levels in the microglia of CLP mice, thereby improving cognitive function^38^. Limited binding of HMGB1 and MD-2 downregulates the expression of NLRP3, thereby alleviating neuroinflammation and cognitive impairment in CLP model mice^39^. Maf1 inhibits proinflammatory cytokine levels and neuronal apoptosis through the NF-κB/NLRP3 inflammasome signaling pathway, thereby improving SAE^40^. Our results showed that MSCs-exo inhibited NLRP3, Caspase 1, and GSDMD-N expression in LPS-induced HMC3 cells and the hippocampal tissue of CLP mice. In vitro and in vivo data showed that MSCs-exo-mediated delivery of miR-140-3p inhibited the neuroinflammatory response and pyroptosis by targeting Hmgb1 and improved cognitive impairment in CLP mice.

Studies have shown that metabolic disorders are related to SAE disease progression^41^. LC–MS metabolomics identified 81 metabolites in the hippocampus of mice with SAE. Integrated pathway analysis revealed that various dysregulated metabolic pathways, including lipid metabolism, amino acid, glucose and nucleotide pathways, inflammation-related pathways, and dysregulated synapses, were closely associated with hippocampal dysfunction in early SAE^42^. Our results showed that the effect of ABX on LPS-induced HMC3 cells was consistent with that of the MSCs-exo plus the miR-140-3p mimic, and ABX had synergistic effects with MSCs-exo plus the miR-140-3p mimic. MSCs-exo plus the miR-140-3p mimic upregulated S-lactoylglutathione levels in the hippocampus of CLP mice. Starch and sucrose metabolism, the pentose phosphate pathway, pyruvate metabolism, cysteine and methionine metabolism, amino sugar and nucleotide sugar metabolism, and fatty acid degradation were associated with hippocampal dysfunction in SAE. The metabolite derivative ethyl pyruvate prevents cognitive impairment in mice with SAE by inhibiting the NLRP3 inflammasome in microglia^43^. Our data further showed that treatment with MSCs-exo plus the miR-140-3p mimic and S-lactoylglutathione increased the GSH levels in LPS-induced HMC3 cells and decreased LD, GLO2, HMGB1, IBA-1, IL-1β, IL-6, iNOS, GSH, TNF-α, NLRP3, Caspase 1, and GSDMD-N levels in LPS-induced HMC3 cells, suggesting that delivery of miR-140-3p by MSCs-exo inhibited the inflammatory response and pyroptosis in LPS-induced HMC3 cells by regulating S-lactoylglutathione metabolism by targeting to Hmgb1.

This study has several limitations. First, the effects of miR-140-3p-mediated delivery of MSCs-exo on other processes in SAE, such as ferroptosis, microglial polarization, and mitochondrial autophagy, need further investigation. In future studies, we will continue to explore the mechanism by which the delivery of miR-140-3p by MSCs-exo affects ferroptosis, microglial polarization, or mitochondrial autophagy in SAE. Second, the delivery of other target molecules of miR-140-3p by MSCs-exo in SAE deserves further analysis. In future projects, we will further clarify the targets and functional mechanisms of MSCs-exo-mediated delivery of miR-140-3p in SAE in combination with RNA sequencing. In addition, the roles of other functional miRNAs within MSCs-exo in SAE need further clarification. Further investigations of other regulatory molecular networks involved in MSCs-exo-mediated improvement in SAE are necessary.

In this study, we showed that the delivery of miR-140-3p by MSCs-exo ameliorated cognitive impairment in CLP mice by improving microglial pyroptosis and S-lactoylglutathione metabolism by targeting Hmgb1. The delivery of miR-140-3p by MSCs-exo might be used for the development of therapeutic strategies.

### Supplementary information

Supplementary information

Description of additional supplementary files

Supplementary Data 1

Reporting Summary

## Data Availability

Uncropped blot images and the source data of the bar/line graphs are included in Supplementary Fig. 1/Supplementary Data 1. Any remaining information can be obtained from the corresponding author upon reasonable request.
