# Supplementary material for: MSC-derived exosomal miR-140-3p improves cognitive dysfunction in sepsis-associated encephalopathy by HMGB1 and S-lactoylglutathione metabolism
Source: Commun Biol. 2024 May 11;7:562. doi: 10.1038/s42003-024-06236-z (PMC11088640; doi:10.1038/s42003-024-06236-z)

Supplementary Methods

Isolation and identification of MSCs

Mouse bone marrow MSCs (BMSCs) were isolated from the bone marrow of the tibia and femur. MSCs were cultured in Dulbecco's modified Eagle's medium (DMEM) supplemented with 100 μg/mL penicillin, 100μg/mL streptomycin, 2-mM glutamine, and 15% fetal bovine serum (FBS) at 37°C and 5% CO_2. Third-generation MSCs were used in this study. The phenotype of MSCs was identified by flow cytometry. In brief, 5×10^5 MSCs were incubated with cluster designation (CD)73-FITC (11-0739-42, eBioscience, USA), CD90-FITC (11-0909-42, eBioscience), CD105-FITC (MA1-19594, eBioscience), CD19-FITC (11-0199-42, eBioscience), CD34-FITC (11-0349-42, eBioscience), CD45-FITC (11-0459-42, eBioscience), and human leukocyte antigen-DR (HLA-DR)-FITC (11-9956-42, eBioscience) antibodies. The proportion of positive cells was examined by flow cytometry (CytoFLEX, Beckman Coulter) and was analyzed by CytExpert software (Version 2.4). Adipogenic differentiation media (Invitrogen, Carlsbad, CA, USA) and osteogenic differentiation media (Invitrogen) were used to differentiate the MSCs. Fourteen days later, the cells were stained with Oil Red O (Sigma-Aldrich, Merck KGaA, Darmstadt, Germany) and Alizarin Red S (Sigma-Aldrich). The miR-140-3p-mimic and negative control (NC)-mimic (RiboBio, Guangzhou, China) were transfected into MSCs using RNAiMAX (Invitrogen).

Extraction of MSCs-exo

After 24 h of transfection, exosomes were isolated from MSCs using total exosome separation reagent (Invitrogen). After 48 h of culture in an exosome-free medium, the MSCs were centrifuged at 500 × g at 4°C for 10 min to ensure the removal of cell fragments and dead cells and were subsequently filtered through a 0.2 mm filter. The exosome extraction reagent and cell culture supernatant were mixed at a 1:5 ratio and incubated at 4°C overnight. The solution was centrifuged at 1500 × g and 4°C for 30 min. The collected exosomes were resuspended in Phosphate-Buffered Saline (PBS) and stored at -80°C for further experiments.

Identification of MSCs-exo

Exosome suspensions (10 μL) were added to a 2 mm Forma-coated copper mesh and incubated at room temperature for 1 min. Exosomes were negatively stained with 3% (w/v) sodium

phosphotungstate solution (Sigma-Aldrich) for 5 min. Then, transmission electron microscopy (TEM, H-8100, Hitachi, Tokyo, Japan) was used to take pictures at 80~120 kV (scare bar = 0.2 μm). A NanoSight NS3000 system (Nanosight, Amesbury, UK) equipped with a blue laser (405 nm) was used for measurements for nanoparticle tracking analysis (NTA).

The uptake of MSCs-exo

According to the manufacturer' instructions, MSCs-exo were labeled with the fluorescent dye PKH67 (Umibio, Shanghai, China). The stained exosomes were then incubated with human microglial clone 3 (HMC3) cells. The cells were stained with 4',6-diamidino-2-phenylindole (DAPI) after 24 h of incubation. Images were captured using a laser scanning confocal microscope (Nikon, Japan).

Terminal-deoxynucleotidyl transferase mediated nick end labeling (TUNEL)

The slices were obtained by immunohistochemical staining. A TUNEL apoptosis detection kit (40306ES50, Yeasen, Shanghai, China) was used to evaluate the proportion of apoptotic cells. The nuclei were stained with DAPI solution at 37°C for 10 min. Glycerin buffer was applied, and the cells were observed under a fluorescence microscope.

Enzyme-linked immunosorbent assay (ELISA)

ELISA kits (CSB-E04741m, CSB-E04740h, CSB-E08054m, CSB-E08053h, CSB-E04639m, CSB-E04638h, CSB-E08223h, CSB-E08148h, CUSABIO) were used. Tumor necrosis factor-α (TNF-α), interleukin (IL)-1β, IL-6, high mobility group box 1 (HMGB1), and inducible nitric oxide synthase (iNOS) levels were detected. The glutathione (GSH) and lactic acid (LD) levels were determined by biochemical kits (A006-2-1, A019-2-1, Nanjing Jiancheng Bioengineering Institute, China).

Liquid chromatography-mass spectrometry (LC-MS)

A mass spectrometer (#TripleTOF5600+, AB Sciex, USA) was used for LC-MS analysis. Hippocampal tissue (3 μL) from the different groups (n = 3) was centrifuged at 1×10^4 rpm for 10 min. The supernatant was collected and diluted with 50% methanol to an appropriate concentration. After the extraction solution mixture was filtered through a 0.22 μm filter, an HSS T3 columm (100 × 2.1 mm, 1.7 μm, Waters, USA) was used at 40°C. The flow rates of solvent A (water containing 0.1% formic acid) and solvent B (acetonitrile containing 0.1%

formic acid) were 0.3 mL/min. The gradient conditions of the liquid phase were as follows: 0.01 min, A: B = 99: 1; 1.5 min, A: B = 99: 1; 13 min, A: B = 1: 99; 16.5 min, A: B = 1: 99; 16.6 min, A: B = 99: 1; and 20 min, stop.

Supplementary Tables

Supplementary Table 1. Primers used for qRT-PCR.

Target gene	Primers	Sequence(5′-3′)
miR-140-3p	Forward	TACCACAGGGTAGAACCACGG
	Reverse	GCTGTCAACGATACGCTACGTAA
miR-4645-5p	Forward	ACCAGGCAAGAAATATTGT
	Reverse	GCTGTCAACGATACGCTACGTA
miR-378b	Forward	ACTGGACTTGGAGGCAGAA
	Reverse	GCTGTCAACGATACGCTACGTA
miR-4275	Forward	CCAATTACCACTTCTTT
	Reverse	GCTGTCAACGATACGCTACGTA
miR-548as-3p	Forward	TAAAACCCACAATTATGTTTGT
	Reverse	GCTGTCAACGATACGCTACGTA
miR-142-3p	Forward	TGTAGTGTTTCCTACTTTATGGA
	Reverse	GCTGTCAACGATACGCTACGTA
miR-450a-5p	Forward	TTTTGCGATGTGTTCCTAATAT
	Reverse	GCTGTCAACGATACGCTACGTA
miR-589-5p	Forward	TGAGAACCACGTCTGCTCTGAG
	Reverse	GCTGTCAACGATACGCTACGTA
miR-147a	Forward	GTGTGTGGAAATGCTTCTGC
	Reverse	GCTGTCAACGATACGCTACGTA
miR-340-5p	Forward	TTATAAAGCAATGAGACTGATT
	Reverse	GCTGTCAACGATACGCTACGTA
U6	Forward	CTCGCTTCGGCAGCACA
	Reverse	AACGCTTCACGAATTTGCGT

Supplementary Table 2. The primary antibody in the study.

Name	Dilution rate	Product code	Manufacturer	Country
p65	1:1000	ab76302	Abcam	UK
p-p65	1:1000	66535-1-Ig	Proteintech	USA
NLRP3	1:800	19771-1-AP	Proteintech	USA
Caspase 1	1:20000	81482-1-RR	Proteintech	USA
Gasdermin D (GSDMD)	1:5000	20770-1-AP	Proteintech	USA
HMGB1	1μg/mL	ab18256	Abcam	UK
GLO2	1:1000	ab154108	Abcam	UK
β-actin	1:5000	66009-1-Ig	Proteintech	USA

Supplementary Figures

Supplementary Figure 1. Uncropped blot images.

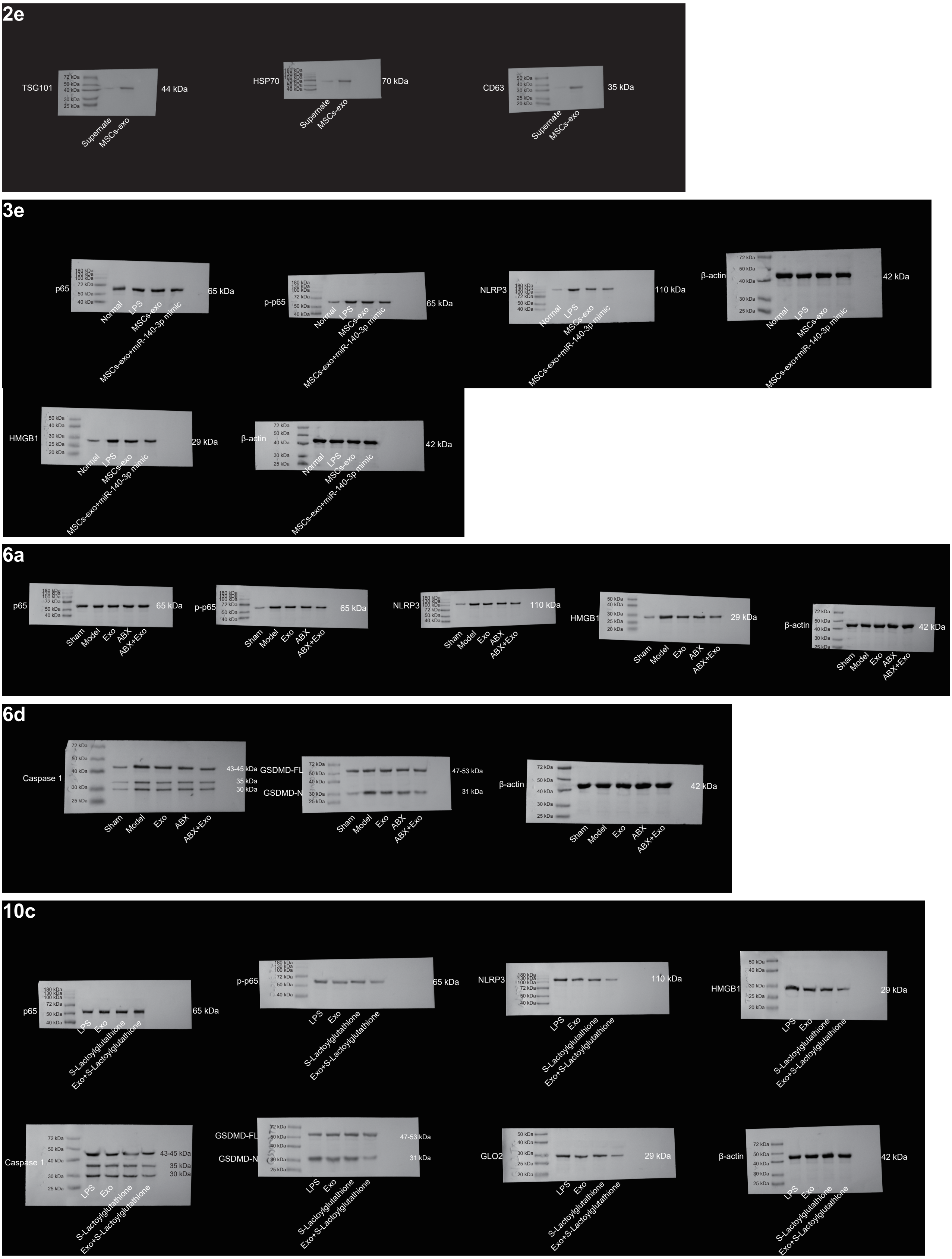

Supplementary Figure 2. Gating strategy of flow cytometry plots.

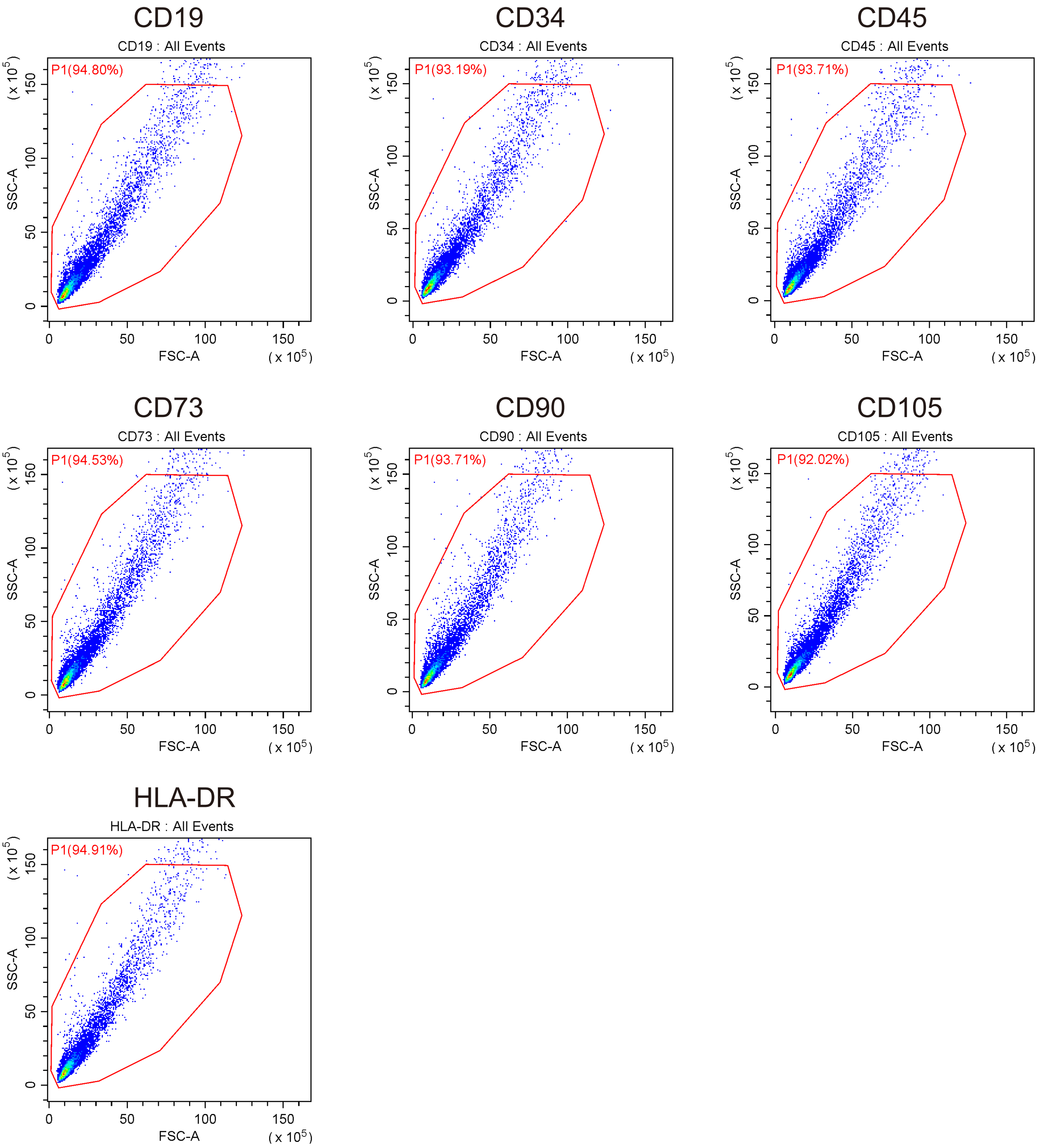

Supplement: Supplementary file 1 — Supplementary information [file 42003_2024_6236_MOESM1_ESM.pdf]
