# Supplementary material for: MSC-derived exosomal miR-140-3p improves cognitive dysfunction in sepsis-associated encephalopathy by HMGB1 and S-lactoylglutathione metabolism
Source: Commun Biol. 2024 May 11;7:562. doi: 10.1038/s42003-024-06236-z (PMC11088640; doi:10.1038/s42003-024-06236-z)
Supplement: Supplementary file 2 — Description of additional supplementary files [file 42003_2024_6236_MOESM2_ESM.docx]

Description of Additional Supplementary Files

**File name:** Supplementary Data 1

**Description:** The source data of the bar graphs and line graphs in this study.
