# Supplementary material for: MSC-derived exosomal miR-140-3p improves cognitive dysfunction in sepsis-associated encephalopathy by HMGB1 and S-lactoylglutathione metabolism
Source: Commun Biol. 2024 May 11;7:562. doi: 10.1038/s42003-024-06236-z (PMC11088640; doi:10.1038/s42003-024-06236-z)
Supplement: Supplementary file 4 — Reporting Summary [file 42003_2024_6236_MOESM4_ESM.pdf]

nature portfolio

Corresponding author(s): XQ COMMSBIO-23-3324

Last updated by author(s): Apr 10, 2024

Reporting Summary

Nature Portfolio wishes to improve the reproducibility of the work that we publish. This form provides structure for consistency and transparency in reporting. For further information on Nature Portfolio policies, see our Editorial Policies and the Editorial Policy Checklist.

Statistics

For all statistical analyses, confirm that the following items are present in the figure legend, table legend, main text, or Methods section.

n/a	Confirmed	
☐	☒	The exact sample size (*n*) for each experimental group/condition, given as a discrete number and unit of measurement
☐	☒	A statement on whether measurements were taken from distinct samples or whether the same sample was measured repeatedly
☐	☒	The statistical test(s) used AND whether they are one- or two-sided<br>*Only common tests should be described solely by name; describe more complex techniques in the Methods section.*
☐	☒	A description of all covariates tested
☐	☒	A description of any assumptions or corrections, such as tests of normality and adjustment for multiple comparisons
☐	☒	A full description of the statistical parameters including central tendency (e.g. means) or other basic estimates (e.g. regression coefficient) AND variation (e.g. standard deviation) or associated estimates of uncertainty (e.g. confidence intervals)
☐	☒	For null hypothesis testing, the test statistic (e.g. *F*, *t*, *r*) with confidence intervals, effect sizes, degrees of freedom and *P* value noted<br>*Give P values as exact values whenever suitable.*
☒	☐	For Bayesian analysis, information on the choice of priors and Markov chain Monte Carlo settings
☒	☐	For hierarchical and complex designs, identification of the appropriate level for tests and full reporting of outcomes
☒	☐	Estimates of effect sizes (e.g. Cohen's *d*, Pearson's *r*), indicating how they were calculated

Our web collection on statistics for biologists contains articles on many of the points above.

Software and code

Policy information about availability of computer code

Data collection	The GSE101639 dataset included three normal controls, three patients with sepsis, and three patients with septic shock and was used to analyze DEmiRNAs associated with SAE. The expression matrix was normalized using the quantile method through the limma package. A |log2FoldChange| > 1 and adjusted P < 0.05 were used to identify DEmiRNAs. TargetScan (http://www.targetscan.org/mamm_31/) was used to predict the miRNAs targeting combination of HMGB1.
Data analysis	The data were analyzed and plotted using GraphPad Prism 9 software. The data are expressed as the mean ± standard deviation (SD). Each test was repeated independently three times from distinct samples. Kolmogorov-Smirnov test and exploratory descriptive statistics test were used to analyze whether the data conformed to a normal distribution and homogeneity of variance. The measurement data obeyed the normal distribution and homogeneity of variance. The data were analyzed by parametric test. The unpaired student's t-test was used to compare the data of two groups that were not one-to-one correspondence using a two-tailed approach. One-way ANOVA and Tukey's post-hoc test were used to compare data among three groups. Data comparisons between groups at different time points were analyzed by two-way ANOVA with Bonferroni as a post hoc test. The significance level was set at P < 0.05.

For manuscripts utilizing custom algorithms or software that are central to the research but not yet described in published literature, software must be made available to editors and reviewers. We strongly encourage code deposition in a community repository (e.g. GitHub). See the Nature Portfolio guidelines for submitting code & software for further information.

Data

Policy information about availability of data

All manuscripts must include a data availability statement. This statement should provide the following information, where applicable:

- Accession codes, unique identifiers, or web links for publicly available datasets
- A description of any restrictions on data availability
- For clinical datasets or third party data, please ensure that the statement adheres to our policy

The data supporting the conclusion are included in the article and the Supplementary information. Any remaining information can be obtained from the corresponding author upon reasonable request.

Research involving human participants, their data, or biological material

Policy information about studies with human participants or human data. See also policy information about sex, gender (identity/presentation), and sexual orientation and race, ethnicity and racism.

Reporting on sex and gender	Not applicable.
Reporting on race, ethnicity, or other socially relevant groupings	Not applicable.
Population characteristics	Not applicable.
Recruitment	Not applicable.
Ethics oversight	Not applicable.

Note that full information on the approval of the study protocol must also be provided in the manuscript.

Field-specific reporting

Please select the one below that is the best fit for your research. If you are not sure, read the appropriate sections before making your selection.

☒ Life sciences ☐ Behavioural & social sciences ☐ Ecological, evolutionary & environmental sciences

For a reference copy of the document with all sections, see nature.com/documents/nr-reporting-summary-flat.pdf

Life sciences study design

All studies must disclose on these points even when the disclosure is negative.

Sample size	The mice were randomly divided into five groups (n = 10): the sham group, model group, MSCs-exo + miR-140-3p mimic (Exo) group, antibiotic (ABX) group, and ABX + Exo group. For liquid chromatography-mass spectrometry (LC-MS) analysis, n=3 hippocampal tissues samples/group.
Data exclusions	No data was excluded.
Replication	All experiments in this study were independently replicated with biological replicates listed in the legends of the corresponding figures
Randomization	Animals were randomly allocated into experimental groups. For in vitro experiments, all samples were completely randomized to treatment groups.
Blinding	The investigators were blinded to group allocation during data collection and analysis of all data presented in the paper.

Reporting for specific materials, systems and methods

We require information from authors about some types of materials, experimental systems and methods used in many studies. Here, indicate whether each material, system or method listed is relevant to your study. If you are not sure if a list item applies to your research, read the appropriate section before selecting a response.

Materials & experimental systems

n/a	Involved in the study	
☐	☒	Antibodies
☐	☒	Eukaryotic cell lines
☒	☐	Palaeontology and archaeology
☐	☒	Animals and other organisms
☒	☐	Clinical data
☒	☐	Dual use research of concern
☒	☐	Plants

Methods

n/a	Involved in the study	
☒	☐	ChIP-seq
☐	☒	Flow cytometry
☒	☐	MRI-based neuroimaging

Antibodies

Antibodies used

For immunohistochemistry and/or immunofluorescence:
Rabbit anti-Caspase 1 (1:300, 22915-1-AP, Proteintech, USA),
Rabbit anti-NLRP3 (1:300, 19771-1-AP, Proteintech, USA),
Mouse anti-HMGB1 (1:200, ab190377, Abcam, UK),
Rabbit anti-IBA-1 (1:50, 10904-1-AP, Proteintech, USA);
HRP-conjugated polyclonal goat anti-rabbit IgG (1:1000, AWS0002, Abiowell, China);
CoraLite488-conjugated Goat Anti-Rabbit IgG(H+L) (1:200, SA00013-2, Proteintech, USA),
CoraLite594-conjugated Goat Anti-Mouse IgG(H+L) (1:200, SA00013-3, Proteintech, USA);

For Western blot:
Rabbit anti-p65 (1:1000, ab76302, Abcam, UK),
Mouse anti-p-p65 (1:1000, 66535-1-Ig, Proteintech, USA),
Rabbit anti-NLRP3 (1:800, 19771-1-AP, Proteintech, USA),
Rabbit anti-Caspase 1 (1:20000, 81482-1-RR, Proteintech, USA),
Rabbit anti-Gasdermin D (GSDMD) (1:5000, 20770-1-AP, Proteintech, USA),
Rabbit anti-HMGB1 (1μg/mL, ab18256, Abcam, UK),
Rabbit anti-GLO2 (1:1000, ab154108, Abcam, UK),
Mouse anti-β-actin (1:5000, 66009-1-Ig, Proteintech, USA);
HRP Goat anti-mouse IgG (1:5000, SA00001-1, proteintech, USA),
HRP Goat anti-rabbit IgG (1:6000, SA00001-2, proteintech, USA);

For flow cytometry:
Anti-CD73-FITC (11-0739-42, eBioscience, USA),
Anti-CD90-FITC (11-0909-42, eBioscience, USA),
Anti-CD105-FITC (MA1-19594, eBioscience, USA),
Anti-CD19-FITC (11-0199-42, eBioscience, USA),
Anti-CD34-FITC (11-0349-42, eBioscience, USA),
Anti-CD45-FITC (11-0459-42, eBioscience, USA),
Anti-HLA-DR-FITC (11-9956-42, eBioscience, USA).

Validation

The validation of each antibody for the species and application is available on the manufacturer's website.

Eukaryotic cell lines

Policy information about cell lines and Sex and Gender in Research

Cell line source(s)

Human microglia HMC3 cells (AW-CNH003, Abiowell, Changsha, China)
HEK293T cells (AW-CNH086, Abiowell, Changsha, China)

Authentication

The cell lines were authenticated by the manufacturers.

Mycoplasma contamination

The cell lines were tested negative for mycoplasma contamination by the manufacturers.

Commonly misidentified lines
(See ICLAC register)

No misidentified cell lines were involved in this study.

Animals and other research organisms

Policy information about studies involving animals; ARRIVE guidelines recommended for reporting animal research, and Sex and Gender in Research

Laboratory animals

C57BL/6J male mice, which aged 6 to 8 weeks and weighed 25-30 g were purchased from Hunan Slack Jingda Laboratory Animal Co., Ltd.

Wild animals

The study did not involve wild animals.

Reporting on sex	male
Field-collected samples	The study did not involve samples collected from the filed.
Ethics oversight	The IRB of Third Xiangya Hospital, Central South University, approved all the experiments in this study (2023-S633).

Note that full information on the approval of the study protocol must also be provided in the manuscript.

Plants

Seed stocks	N/A
Novel plant genotypes	N/A
Authentication	N/A

Flow Cytometry

Plots

Confirm that:

☒ The axis labels state the marker and fluorochrome used (e.g. CD4-FITC).

☒ The axis scales are clearly visible. Include numbers along axes only for bottom left plot of group (a 'group' is an analysis of identical markers).

☒ All plots are contour plots with outliers or pseudocolor plots.

☒ A numerical value for number of cells or percentage (with statistics) is provided.

Methodology

Sample preparation	Mouse bone marrow MSCs (BMSCs) were isolated from the bone marrow of the tibia and femur. MSCs were cultured in Dulbecco's modified Eagle's medium (DMEM) supplemented with 100 μg/mL penicillin, 100μg/mL streptomycin, 2-mM glutamine, and 15% fetal bovine serum (FBS) at 37℃ and 5% CO2. Third-generation MSCs were used in this study. The phenotype of MSCs was identified by flow cytometry.
Instrument	CytoFLEX, Beckman Coulter
Software	CytExpert software (Version 2.4)
Cell population abundance	No flow cytometry cell sorting operation was involved in this study.
Gating strategy	Several gating steps were performed consecutively: FSC-A/SSC-A (single cells); CD19+, CD34+, CD45+, CD73+, CD90+, CD105 +, HLA-DR+(baseline set based on the non-fluorescent control strain).

☒ Tick this box to confirm that a figure exemplifying the gating strategy is provided in the Supplementary Information.
